# Insuficiência Mitral e Troca Valvar Aórtica Transcateter: Há Outras Implicações Prognósticas?

**DOI:** 10.36660/abc.20210392

**Published:** 2021-06-08

**Authors:** Antonio de Santis

**Affiliations:** 1 Universidade de São Paulo Instituto do Coração Unidade Clinica de Valvopatia São PauloSP Brasil Universidade de São Paulo Instituto do Coração - Unidade Clinica de Valvopatia , São Paulo , SP - Brasil

**Keywords:** Insuficiência da Valva Mitral/cirurgia, Idoso, Morbimortalidade, Substituição de Valva Aórtica Transcateter

Há uma prevalência variável de regurgitação mitral moderada a grave (13 a 74%) em pacientes com estenose aórtica degenerativa grave. ^1 - 6^ Em pacientes idosos e frágeis, essa associação pode gerar dilemas clínicos na prática cardiológica: devo submeter meu paciente a cirurgia valvar combinada, com exposição a maior morbimortalidade, ou contemplar apenas a estenose aórtica grave com um tratamento menos agressivo representado pela troca valvar aórtica transcateter (TAVR, do inglês *transcatheter aortic valve replacement* )? Os graus variáveis de gravidade, aliados à etiologia comumente funcional (até 80%) da regurgitação mitral associada, colocam a estenose da válvula aórtica em uma posição de destaque nesta hierarquia clínica com uma predileção resultante para realização da TAVR nesses cenários. De fato, alguns estudos apresentaram melhora na gravidade da regurgitação mitral após a TAVR, principalmente em pacientes com etiologia funcional e sem hipertensão pulmonar ou fibrilação atrial. ^5 - 7^

O impacto da regurgitação mitral basal em pacientes submetidos a TAVR ainda é controverso. Toggweiler et al., ^7^ descobriram que a regurgitação mitral basal moderada a grave em pacientes submetidos a TAVR estava associada a taxas mais altas de mortalidade precoce (primeiros 30 dias), sem diferença na mortalidade tardia. ^7^ Pelo contrário, Barbanti et al., ^8^ utilizando dados da coorte A do estudo randomizado *Placement of Aortic Transcatheter Valve* (PARTNER), descobriram que a regurgitação mitral basal moderada a grave estava associada a mortalidade tardia mais alta apenas no grupo de substituição cirúrgica da válvula aórtica, sem implicação prognóstica no grupo TAVR. ^8^

Um aspecto muito relevante e ainda pouco explorado é o valor prognóstico das alterações no grau de gravidade da regurgitação mitral após a TAVR. Nesse contexto, o presente estudo conduzido por Cunha et al. ^9^ consolida o papel da regurgitação mitral como marcador prognóstico após a TAVR, enfatizando que a piora prospectiva da regurgitação mitral é um preditor independente de mortalidade no período pós-TAVR. ^9^ Ao utilizar o registro brasileiro de TAVR, os autores tiveram acesso a dados de 22 centros nacionais, permitindo a inclusão de 795 pacientes para análise. Dentre os selecionados, 19,3% apresentavam insuficiência mitral basal moderada a grave associada a estenose aórtica grave. Os preditores independentes relatados de mortalidade tardia (seguimento médio de 16,6 meses) foram: doença vascular periférica, valvuloplastia aórtica por balão anterior e insuficiência mitral basal moderada a grave, como demonstrado em estudos anteriores.

Houve melhora da regurgitação mitral em quase 50% dos pacientes com refluxo moderado a grave, enquanto uma pequena parcela dos pacientes apresentou piora da regurgitação mitral (8,7%). Esse achado, assim como as evidências anteriores, reforça que a TAVR, ao reduzir as pressões de enchimento ventricular e restaurar um fluxo adequado na via de saída do ventrículo esquerdo, pode determinar uma redução no volume regurgitante mitral. ^6 - 8^ Curiosamente, os autores descobriram que os pacientes com melhora na regurgitação mitral no período pós-TAVR apresentaram uma fração de ejeção basal mais baixa. Nesses casos, a influência da TAVR na redução do volume diastólico final e na remodelação ventricular reversa pode favorecer a geometria do anel valvar mitral, principalmente nas etiologias funcionais. Infelizmente, não foi possível determinar a etiologia da regurgitação mitral na população estudada. Além disso, um dos achados mais reveladores do presente estudo foi o impacto negativo da piora da gravidade da insuficiência mitral no seguimento tardio desses pacientes, levando à maior mortalidade representada pelas curvas específicas de Kaplan-Meier.

O agravamento da regurgitação mitral no período pós-TAVR poderia ser outro potencial preditor de desfecho, reforçando a importância da monitoração ecocardiográfica periódica durante o acompanhamento clínico desses pacientes. Essa progressão também sugere que as abordagens complacentes usuais, como as terapias farmacológicas, podem não ser suficientes para evitar desfechos negativos. Possivelmente, o uso de estratégias de correção percutânea para insuficiência mitral grave no período pós-TAVR poderia ser mais explorado, sempre com base em decisões colegiadas por equipes cardíacas institucionais.
