# Supplementary material for: Insuficiência Mitral e Troca Valvar Aórtica Transcateter: Há Outras Implicações Prognósticas?
Source: Arq Bras Cardiol. 2021 Jun 8;116(6):1070–1. [Article in Portuguese] doi: 10.36660/abc.20210392 (PMC8288546; doi:10.36660/abc.20210392)
Supplement: Translation

# Translation: Mitral Regurgitation and Transcatheter Aortic Valve Replacement: Are There Any Other Prognostic Implications?

**Authors:** Antonio de Santis

**Affiliations:** 1 Brazil Universidade de São Paulo Instituto do Coração - Unidade Clinica de Valvopatia, São Paulo, SP - Brazil

**Keywords:** Mitral Valve Insufficiency/surgery, Elderly, Morbimortality, Transcatheter Aortic Valve Replacement

There is a variable prevalence of moderate to severe mitral regurgitation (13 to 74%) in patients with severe degenerative aortic stenosis.^1 - 6^ In elderly and frail patients, this association can generate clinical dilemmas in cardiological practice: should I submit my patient to combined valve surgery, exposing him to higher morbidity and mortality, or contemplate only the severe aortic stenosis with a less aggressive treatment represented by the transcatheter aortic valve replacement (TAVR)? The variable degrees of severity associated with the commonly functional etiology (up to 80%) of the associated mitral regurgitation, places aortic valve stenosis in a high prominent position at this clinical hierarchy, with a resultant predilection for performing TAVR in these scenarios. In fact, some studies showed an improvement in the mitral regurgitation severity after TAVR, especially in patients with functional etiology and without pulmonary hypertension or atrial fibrillation.^5 - 7^

The impact of baseline mitral regurgitation on patients undergoing TAVR is still controversial. Toggweiler et al.^7^ found that moderate to severe baseline mitral regurgitation in patients undergoing TAVR was associated with higher early mortality rates (first 30 days), with no difference in late mortality.^7^ Conversely, Barbanti et al.,^8^ using data from the randomized Placement of Aortic Transcatheter Valve (PARTNER) trial cohort A, found that moderate to severe baseline mitral regurgitation was associated with higher late mortality only in the surgical aortic valve replacement group, with no prognostic implication in the TAVR group.^8^

A very relevant and still little explored aspect is the prognostic value of changes in the degree of mitral regurgitation severity after TAVR. In this context, the present study conducted by Cunha et al.^9^ consolidates the role of mitral regurgitation as a prognostic marker after TAVR, emphasizing that the prospective worsening of mitral regurgitation is an independent mortality predictor in the post-TAVR period.^9^ By using the Brazilian TAVR registry, the authors had access to data from 22 national centers, allowing the inclusion of 795 patients for the analysis. Among those selected, 19.3% had moderate to severe baseline mitral regurgitation associated with severe aortic stenosis. The reported independent predictors of late mortality (mean follow-up period of 16.6 months) were: peripheral vascular disease, previous aortic balloon valvuloplasty and moderate to severe baseline mitral regurgitation, as demonstrated in previous studies.

Mitral regurgitation improvement was observed in almost 50% of the patients with moderate to severe reflux, while a small portion of patients showed mitral regurgitation worsening (8.7%). This finding, as well as the previous evidence, reinforces that TAVR, by reducing ventricular filling pressures and restoring an appropriate flow in the left ventricular outflow tract, can determine a reduction in the mitral regurgitant volume.^6 - 8^ Interestingly, the authors found that patients with mitral regurgitation improvement in the post-TAVR period had a lower baseline ejection fraction. In these cases, the influence of TAVR in the reduction of the final diastolic volume and in the reverse ventricular remodeling may favor the geometry of the mitral valve annulus, especially in functional etiologies. Unfortunately, it was not possible to determine the mitral regurgitation etiology in the studied population. Furthermore, one of the most revealing findings of the present study was the negative impact of the worsening of the mitral regurgitation severity in the late follow-up of these patients, leading to higher mortality represented by the specific Kaplan-Meier curves.

The mitral regurgitation worsening in the post-TAVR period could be another potential outcome predictor, reinforcing the importance of periodic echocardiographic monitoring during the clinical follow-up of these patients. This progression also suggests that the usual lenient approaches, such as pharmacological therapies, may not be enough to prevent negative outcomes. Possibly, the use of percutaneous correction strategies for severe mitral regurgitation in the post-TAVR period could be further explored, always based on collegial decisions through institutional heart teams.
